# Chicken feather hydrolysate as alternative peptone source for microbial cultivation

**DOI:** 10.12688/f1000research.17134.3

**Published:** 2019-08-07

**Authors:** Oghenerobor B. Akpor, Damilola E. Odesola, Remilekun E. Thomas, Olarewaju M. Oluba

**Affiliations:** 1 Department of Microbiology, Landmark University, Omu-Aran, Kwara, 251101, Nigeria; 2 Department of Biochemistry, Landmark University, Omu-Aran, Kwara State, 251101, Nigeria

**Keywords:** Culture media, chicken feather, keratin, hydrolysate, bacteria, yeasts, growth rate, protein source

## Abstract

**Background:** Commercially available conventional growth media for the culture of microbes are expensive, hence the need for alternative cheaper sources. Livestock waste, in the form of feather and blood, are of value in biotechnology because of their high protein content. Hence the primary aim of this study was to produce a cheaper peptone alternative from chicken feather protein hydrolysate (CFPH) and blood meal (BM).

**Methods:** The growth of selected bacteria and fungi was monitored in different media prepared from varied concentrations of peptone, CFPH and BM in order to determine the combination that produced maximum growth. Five different media, namely 100% peptone (control), 100% BM, 40% peptone + 60% CFPH, 40% BM + 60% CFPH and 20% peptone + 20% BM + 60% CFPH were prepared and used for the study. The different media were inoculated with 1 ml of each test organism ( *Escherichia coli*, *Klebsiella pneumoniae*, *Proteus mirabilis*, *Staphylococcus aureus, Pseudomonas aeruginosa, Candida carpophila*, *Candida tropicalis* and *Pichia kundriavzevii*) and their growth monitored for 10 h.

**Results:** *Pseudomonas aeruginosa*, *Proteus mirabilis* and *Staphylococcus aureus* grew best in the 100% peptone, *Klebsiella pneumoniae* grew best in 100 BM. The fungi species were observed to grow best in 100% peptone. The 60% CFPH + 40% peptone combination (CFPH obtained with precipitate of trichloroacetic acid (TCA), hydrochloric acid (HCl) and nitric acid (HNO _3_) gave the best growth of *E. coli*.  The 60% CFPH + 40% peptone combination (CFPH obtained with precipitate of TCA) also gave the best growth of *C. tropicalis* and *Klebsiella pneumoniae*.

**Conclusions:** Overall, the 60% CFPH + 40% peptone combination showed the most potential as an alternative to peptone, especially for *E. coli*.

## Introduction

Microbial culture media is composed of different nutrients required by organisms for growth. The nutrient requirements of microorganisms differ from one to another as there are many types of microorganisms. Generally, the microbial growth composition includes carbon and energy sources, protein hydrolysates, otherwise known as peptones, extracts, buffers and sometimes gelling agents. Microorganisms will only be able to grow if they are provided with the appropriate nutrients for growth. Apart from carbon, microbes require a source of nitrogen. Amino acids, urea, ammonia and other compound may serve as the nitrogen source. Some organisms also possess the ability to metabolize peptides and more complex proteins ( Sandle, 2016).

The protein source for microbial culture is derived from peptone, which is a good source of amino acids, peptides and proteins in growth media. It is an excellent source of nitrogen. However, it is also a very expensive constituent of the microbial culture media ( Taskin & Kubanoglu, 2011). Different natural products, such as milk, animal tissues and plants, are being exploited for obtaining peptone in order to reduce the costs of production of the growth media ( Jayathilakan *et al*., 2012; Ben Rebah & Miled, 2013).

It is estimated that about 20 million tons of chicken feathers are generated weekly worldwide and are considered menaces in terms of solid waste pollution ( Egelyng *et al*., 2018; Tesfaye *et al*., 2018). In a bid to dispose of the large amounts waste chicken feathers generated worldwide, methods such as landfill and burning have been used, which have taken a toll on the environment ( Sharma *et al*., 2017).

To reduce the burden of waste generated by disposed chicken feathers, a number of processes and operations involving the application of chicken feathers have been reported. Chicken feathers have been employed in the production of animal feed, textile production and paper production amongst many others ( Chinta *et al*., 2013; Moritz & Latshaw, 2001; Tesfaye *et al*., 2017).

Chicken feathers contain more than 90% protein (keratin), 1% lipids and 8% water ( Lasekan *et al*., 2013). Keratin proteins are grouped into the alpha and beta keratins. Chicken feathers and feathers from most birds are composed majorly of the beta-keratin. Keratin contains all 20 amino acids linked together by peptide bonds, which include covalent disulphide bonds, ionic bonds, hydrogen bonds and hydrophobic bonds ( Greenwold *et al*., 2014). Keratins are bonded by a number of these bonds which make them naturally insoluble. These bonds require that they be broken in other to obtain the chicken feathers in usable forms for microorganisms. By hydrolysis, the bonds are broken, a soluble product is formed (hydrolysate) and the peptone can be obtained from the chicken feather keratin ( Ayutthaya & Wootthikanokkhan, 2013). The conversion of such large amounts of chicken feathers into hydrolyzed forms for the manufacture of microbial culture media can be used as a measure of solving this problem. The chicken feather keratin can then be incorporated into the production of microbial culture media. This study was therefore targeted at utilizing the keratin in the waste chicken feathers as a cheaper alternative to peptone and also a nitrogen source for microbial growth.

## Methods

### Pretreatment of chicken feathers

Chicken feathers were collected from the poultry house in Landmark University Commercial Farm in Omu-Aran, Kwara State, Nigeria. The feathers were first washed with water and laundry detergent before disinfecting with 5% hypochloride solution, as described previously by Akpor *et al.* (2018a). Following disinfection, the feathers were sun-dried for one week and stored in baskets until when needed.

### Feather hydrolysis

Hydrolysis of the feather carried out as reported by earlier investigators ( Akpor *et al.*, 2018a). For hydrolysis, approximately 400 g of dried chicken feathers were placed in 10-l plastic container, after which 2000 ml 1 M NaOH was added. The feather-NaOH mixture was stirred vigorously and left to stand for 10 h. After hydrolysis, the mixture was filtered with a clean, dry muslin cloth and the unhydrolyzed fraction estimated. The quantification of the unhydrolyzed fraction was carried out after drying in an oven to constant weight and then weighed to ascertain the degree of hydrolysis.

### Precipitation of feather keratin

Feather keratin was precipitated separately from the hydrolyzed feather solution, using 1 M solutions of the following acids: hydrochloric acid (HCl), sulphuric acid (H _2_SO _4_), trichloroacetic acid (TCA) and nitric acid (HNO _3_). This was carried out for 10 min at 25°C. The choice of the acids and concentration of NaOH was based on the findings during the method development stage of the study. During this stage several acids and different concentrations of the NaOH were tested, of which 1 M NaOH was ascertained to be the lowest concentration that gave maximum hydrolysis and while the acids gave the highest yield among the organic and inorganic acids that were used for precipitation.

The precipitated chicken feather hydrolysate was separated from the solution by filtration. The chicken feather hydrolysate of the respective acids was air-dried and quantified. The chicken feather hydrolysate of the respective acids (henceforth referred to as feather keratin or hydrolysate) were ground into fine powders using a laboratory blender.

### Preparation of blood meal

Fresh cattle blood was obtained from the abattoir of Landmark University, Omu-Aran (Nigeria) Teaching and Research Farm. Blood meal was prepared using a modification of the protocol described by Amu *et al*., 2018. The blood was immediately heated at 60°C for 30 min to coagulate. The coagulated blood was then placed in aluminum foil and oven-dried at 50°C for 8 h. The dried coagulated blood was milled into powder, using a mechanical grinder. The powdered blood, referred to as blood meal (BM) was kept in air-tight container until required.

### Chicken feather hydrolysate liquid growth media

For this study, different compositions of feather keratin, peptone (Oxoid, UK) and BM were used in the media formulations. Five different combinations were used to make the respective growth media, as follows. Combination A: 60% keratin + 40% peptone + 0% BM; Combination B: 60% keratin + 20% peptone + 20% BM; Combination C: 60% keratin + 0% peptone + 40% BM; Combination D: 100% peptone; Combination E: 100% BM.

The respective components were weighed separately and dissolved in aliquot quantities of distilled water before combining all the components together to make a final media concentration of 5 g/l. Each media, as composed, was dispensed in conical flask and sterilized in an autoclave (121°C for 15 min) at 15 psi.

### Test microbial species

A total of eight microbial species, consisting of five bacteria and three yeast species were used for the study. The bacteria species were *Escherichia coli*, *Klebsiella pneumoniae*, *Proteus mirabilis*, *Staphylococcus aureus* and *Pseudomonas aeruginosa.* The yeasts were *Candida carpophila*, *Candida tropicalis* and *Pichia kundriavzevii*. All the isolates were part of the laboratory stocks in the Department of Microbiology, Landmark University, Nigeria.

Prior to use, stock microbial cultures of the isolates were first streaked on agar plates to ascertain their purity before subculturing into nutrient broth.

### Microbial growth studies

For growth studies, the flasks containing the sterile media were inoculated with the test microbial species. Equal volumes of broth cultures of the pure isolates (0.5 ml) were used for inoculation. The bacteria and yeast cultures were incubated at 37±2°C. and 25±2°C in shaking incubators at shaking speed of 100 rpm.

Following inoculation and every 2 h, for a 10 h duration, aliquot samples were withdrawn from each flask to monitor growth by measuring absorbance using a spectrophotometer at a wavelength of 750 nm, using sterile nutrient broth to normalize. Each experiment was carried out in duplicate.

### Statistical analysis

Data analysis was carried out using the SPSS statistical software, version 13.0. Comparison of means was determined using the one-way ANOVA test at a significance level of P<0.05. Post hocs test was carried out using the least-significance-difference (LSD). All experimental setups were in duplicates.

## Results

Raw absorbance values for each microbe are available on figshare ( Akpor *et al*., 2018b).

### Growth rate of *Candida carpophila*

**Figure 1.**
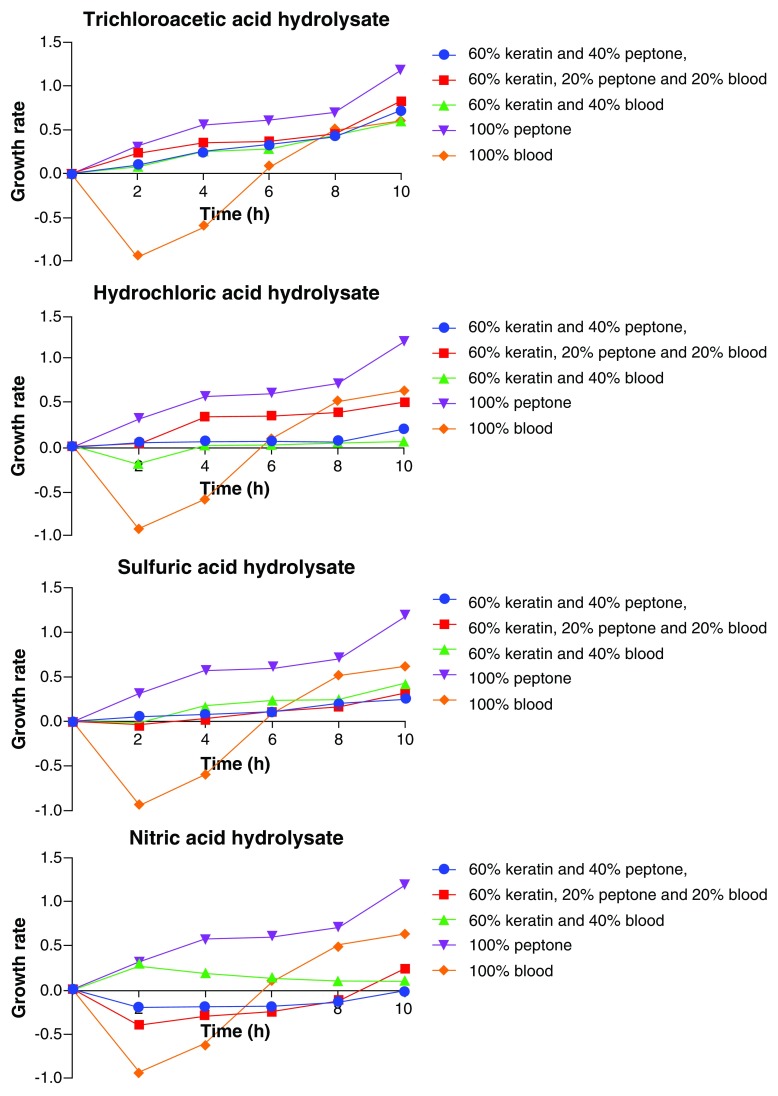
Growth rate of *Candida carpohila* in media with the TCA, HCl, H _2_SO _4_ and HNO _3_ hydrolysates.

As shown in Figure 1, the growth pattern of *Candida carpophila* in all the acid hydrolysate media showed consistent increase with time. The highest growth was observed in the media with 100% peptone. This trend was irrespective of the acid hydrolysate used. Besides, the 100% peptone media, the ranking of the growth of the organism from the highest to the least in the other media compositions varied for each acid feather hydrolysate. In the TCA feather hydrolysate media, after 10 hours, the organism recorded the highest growth in the 100% peptone media, closely followed by 60% feather hydrolysate + 20% peptone + 20% BM, 60% feather hydrolysate + 40% peptone, and 100% BM in that order, while the least growth was observed with 60% feather hydrolysate + 40% BM ( Figure 1).

For the HCl feather hydrolysate media, the growth pattern observed followed the order: 100% peptone > 100% BM > 60% feather hydrolysate + 20% peptone + 20% BM > 60% feather hydrolysate + 40% peptone, while the 60% feather hydrolysate + 40% BM media showed the least growth. When H _2_SO _4_ feather hydrolysate media was used, the growth was in the order: 100% peptone > 100% BM > 60% feather hydrolysate + 40% BM > 60% feather hydrolysate + 20% peptone + 20% BM, while the 60% feather hydrolysate + 40% peptone was least. In the HNO _3_ feather hydrolysate media, *C. carpophila* grew in the order 100% peptone > 100% BM > 60% feather hydrolysate + 20% peptone + 20% BM > 60% feather hydrolysate + 40% BM, and the lowest growth was recorded in the 60% feather hydrolysate + 40% peptone ( Figure 1).

### Growth rate of the *Candida tropicalis*

**Figure 2.**
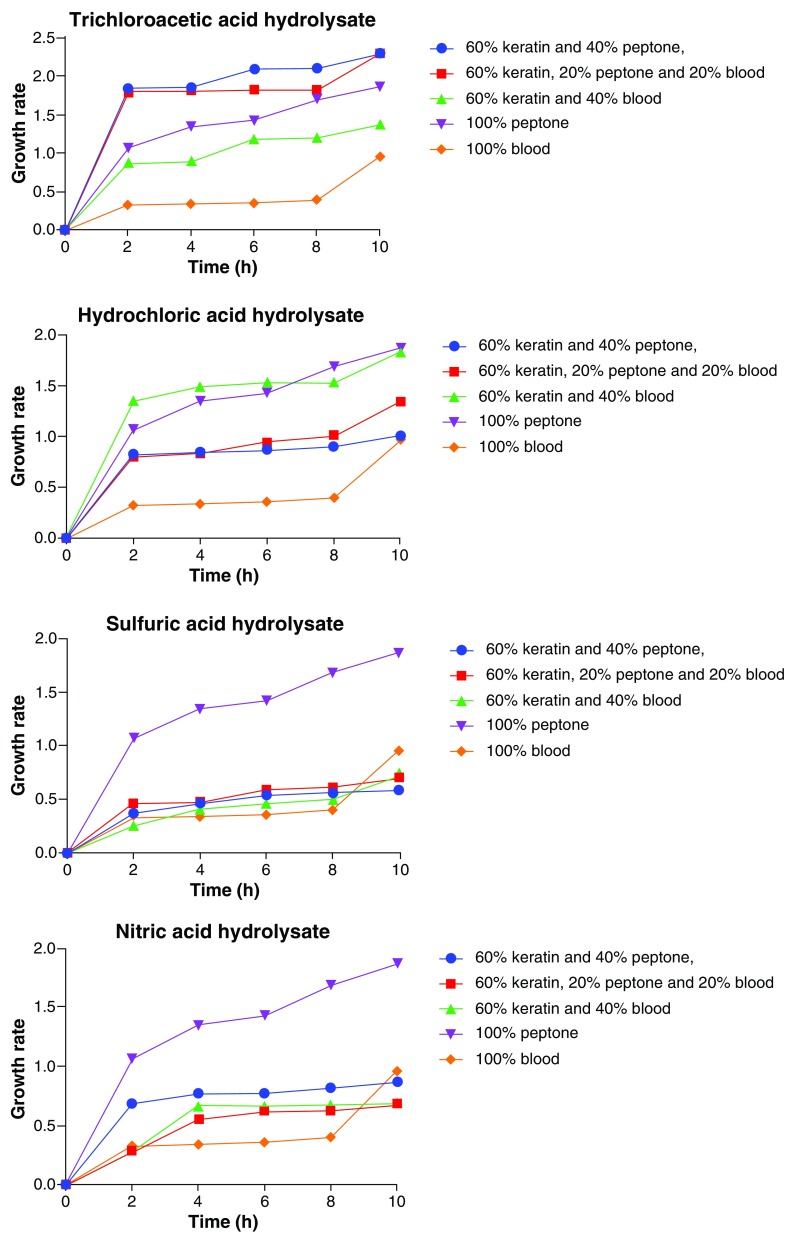
Growth rate of *Candida tropicalis* in media with the TCA, HCl, H _2_SO _4_ and HNO _3_ hydrolysates.

As represented in Figure 2, the highest and the lowest growth of *Candida tropicalis* varied with media composition for each of the acid feather hydrolysates used. However, the most frequent media composition in which the highest growth of the organism was recorded was 100% peptone and the organism showed consistent increase in growth throughout the 10-h period with the highest growth at the 10 ^th^ hour. When the trichloroacetic acid feather hydrolysate media was used, *C. tropicalis* showed no significant difference (p > 0.05) in growth with both 60% feather hydrolysate + 20% peptone + 20% BM and 60% feather hydrolysate + 40% peptone and the organism’s growth was observed to be highest with both compositions. This was followed by 100% peptone, 60% feather hydrolysate + 40% BM and the least growth was with 100% BM ( Figure 2).

In the HCl feather hydrolysate media, the growth of *C. tropicalis* was highest with 100% peptone followed by 60% feather hydrolysate + 40% BM, 60% feather hydrolysate + 20% peptone + 20% BM, 60 feather hydrolysate+ 40% peptone, with the lowest growth occurring in the 100% BM. For the H _2_SO _4_ feather hydrolysate media, the highest growth of *C. tropicalis* was observed with 100% peptone, followed by 100% BM, 60% feather hydrolysate + 40% BM, 60% feather hydrolysate + 20% peptone + 20% BM and the least growth was observed with 60% feather hydrolysate+ 40% peptone. In the HNO _3_ hydrolysate media, *C. tropicalis* had its highest growth with 100% peptone, followed by 100% BM, 60% feather hydrolysate + 40% peptone, 60% feather hydrolysate + 40% BM, and had its least growth with 60% feather hydrolysate + 20% peptone + 20% BM ( Figure 2).

### Growth rate of the *Escherichia coli*

**Figure 3.**
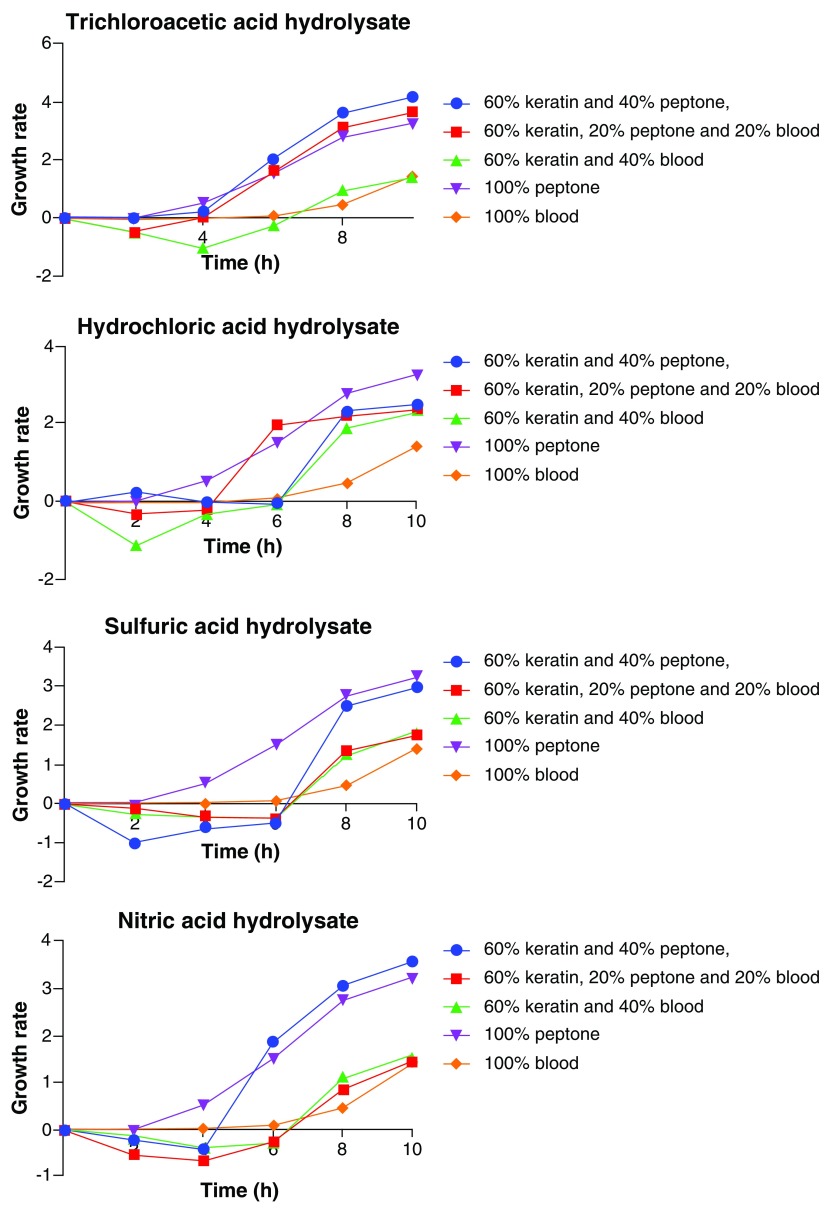
Growth rate of *E. coli* in media with the TCA, HCl, H _2_SO _4_ and HNO _3_ hydrolysates.

As illustrated in Figure 3, the *E. coli* showed consistent increase in growth with the highest growth at the 10 ^th^ hour. The highest and lowest growths of the organism varied with media composition for all the acid feather hydrolysates. In the TCA feather hydrolysate media, the highest growth of *E. coli* at the end of the 10 h period was observed with 60% feather hydrolysate + 40% peptone, followed by 60% feather hydrolysate + 20% peptone + 20% BM, 100% peptone while the least growth was observed with 100% BM and 60% feather hydrolysate + 40% BM. There was no significant difference (p > 0.05) in growth of the organism in both media compositions. When the HCl feather hydrolysate media was used, highest growth of the *E. coli* was with 100% peptone, followed by 60% feather hydrolysate+ 40% peptone, 60% feather hydrolysate + 20% peptone + 20% BM and 60% feather hydrolysate + 40% BM while the least growth was with 100% BM ( Figure 3).

For the H _2_SO _4_ feather hydrolysate media, *E. coli* had its highest growth with 100% peptone followed by 60% feather hydrolysate + 40% peptone, and then 60% feather hydrolysate+ 40% BM and 60% feather hydrolysate + 20% peptone + 20% BM in which there was little or no significant difference in growth while the least growth of *E. coli* was recorded with 100% peptone. In the HNO _3_ feather hydrolysate media, the highest growth of *E. coli* recorded was with 60% feather hydrolysate+ 40% peptone, followed by 100% peptone, while the least growths were observed with 60% feather hydrolysate + 40% BM, 60% feather hydrolysate + 20% peptone + 20% BM and 100% BM, all showing no significant difference (p > 0.05) in growth ( Figure 3).

### Growth rate of the *Klebsiella pneumoniae*

**Figure 4.**
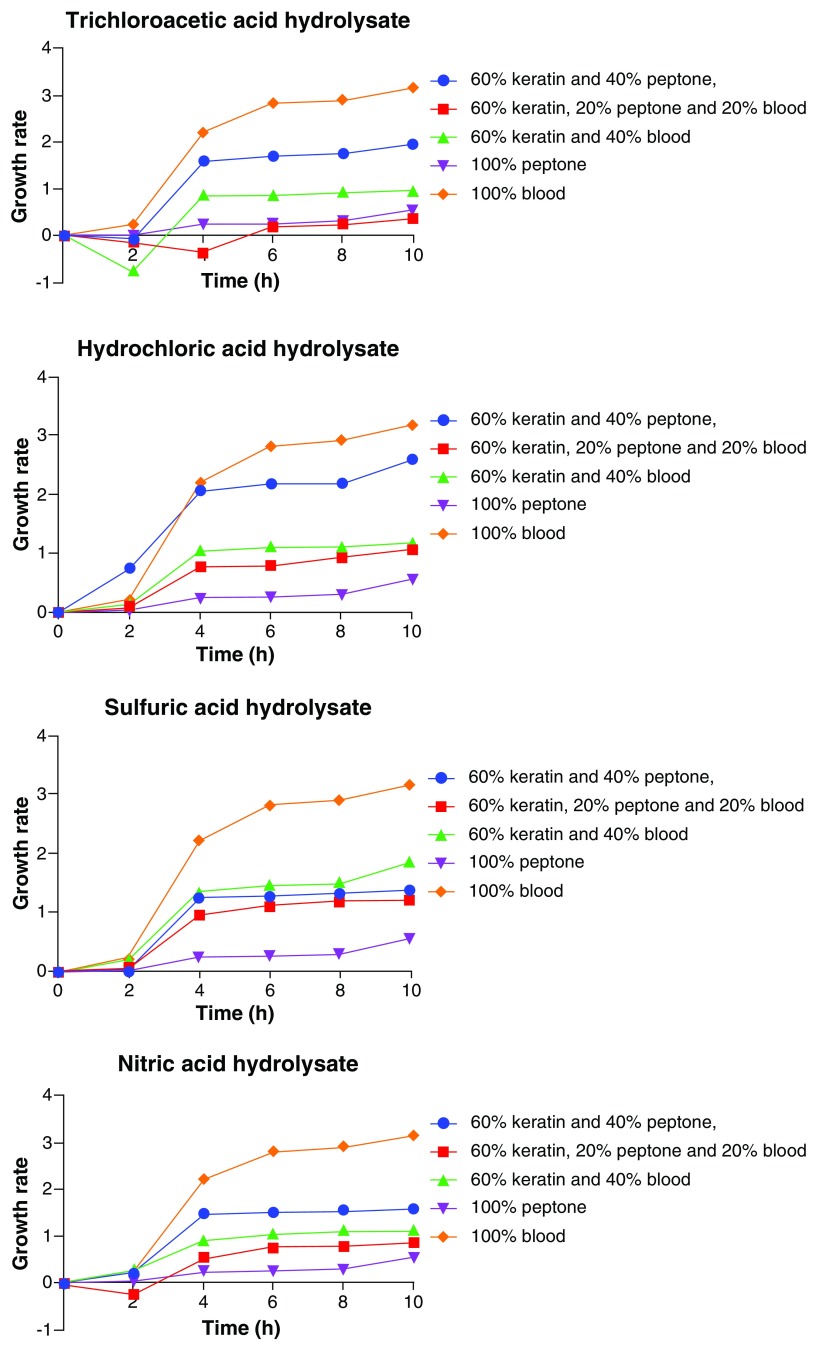
Growth rate of *Klebsiella pneumoniae* in media with the TCA, HCl, H _2_SO _4_ and HNO _3_ hydrolysates.

The results in Figure 4 showed consistent increase in growth rate of *Klebsiella pneumoniae* throughout the 10 h period with highest growth at the 10 ^th^ hour. The highest and lowest growths of the organism varied with the media composition. In the trichloroacetic acid feather hydrolysate media, the highest growth of *Klebsiella pneumoniae* was with 100% BM, followed by 60% feather hydrolysate + 40% peptone, 60% feather hydrolysate + 40% BM, 100% peptone, and the least growth was with 60 feather hydrolysate + 20% peptone + 20% BM. In the HCl feather hydrolysate media, the *Klebsiella pneumoniae* had its highest growth with 100% BM, followed by 60% feather hydrolysate+ 40% peptone, 60% feather hydrolysate + 40% BM, 60% feather hydrolysate + 20% peptone + 20% BM, and the least growth was with 100% peptone ( Figure 4).

In the H _2_SO _4_ feather hydrolysate media, composition in which *Klebsiella pneumoniae* had its highest growth was 100% BM, followed by 60% feather hydrolysate + 40% BM, 60% feather hydrolysate + 40% peptone and then, 60% feather hydrolysate + 20% peptone + 20% BM, while the least was recorded in 100% peptone media. In the HNO _3_ feather hydrolysate media, the highest growth of *Klebsiella* was observed in 100% BM, followed by 60% feather hydrolysate + 40% peptone, 60% feather hydrolysate + 40% BM, 60% feather hydrolysate+ 20% peptone + 20% BM and the least growth of the organism was observed in 100% peptone ( Figure 4).

### Growth rate of the *Pseudomonas aeruginosa*

**Figure 5.**
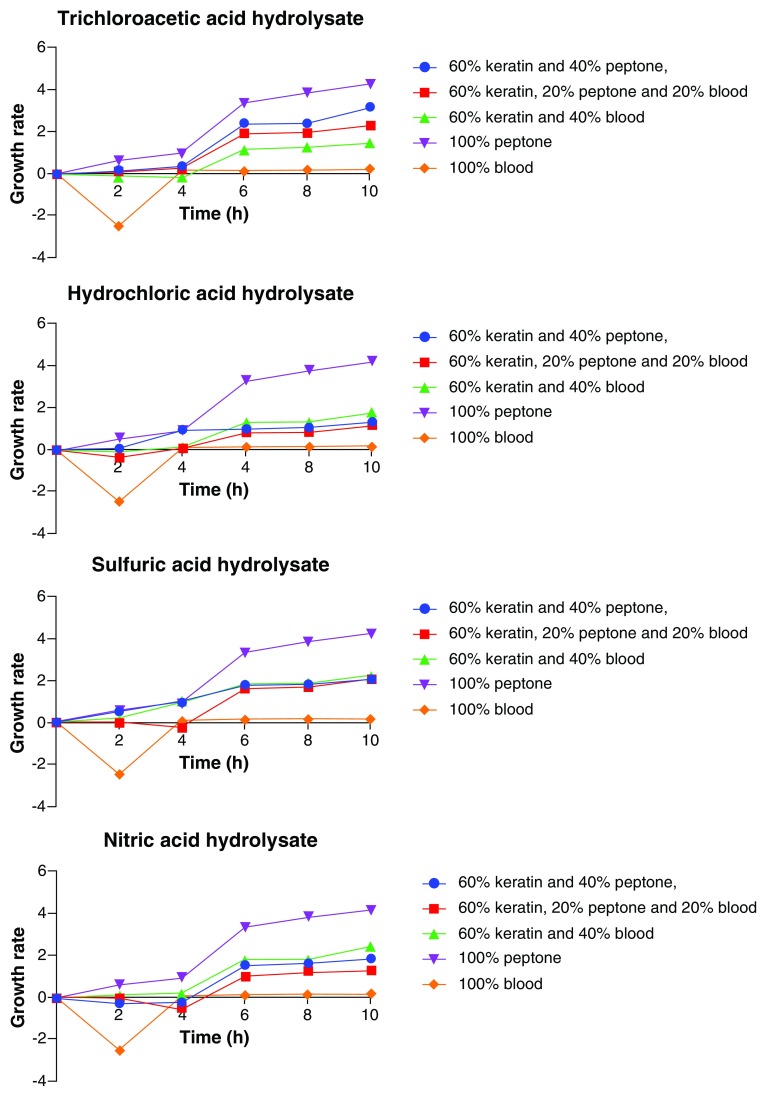
Growth rate of *Pseudomonas aeruginosa* in media with the TCA, HCl, H _2_SO _4_ and HNO _3_ hydrolysates.

As represented in Figure 5, *Pseudomonas aeruginosa* showed consistent increase in growth for the 10 h period and the highest growth was recorded at the 10 ^th^ hour. The media composition which yielded the highest growth of the organism was 100% peptone and the least growth was recorded with 100% BM. This was constant for all the acid hydrolysate media. In the TCA feather hydrolysate media, the media composition after 100% peptone that yielded the highest growth of *P. aeruginosa* was 60% feather hydrolysate+ 40% peptone, followed by 60% feather hydrolysate + 20% peptone + 20% BM, 60% feather hydrolysate + 40% BM, while the least growth of *Pseudomonas aeruginosa* was with 100% BM ( Figure 5).

In the HCl feather hydrolysate media, after 100% peptone, *P. aeruginosa* had its highest growth with 60% feather hydrolysate + 40% BM, followed by 60% feather hydrolysate + 40% peptone, 60% feather hydrolysate + 20% peptone + 20% BM, while the organism had its least growth with 100% BM. In the H _2_SO _4_ feather hydrolysate media, 100% peptone remained the media composition in which the highest growth of *P. aeruginosa* was recorded, followed by 60% feather hydrolysate + 40% BM, 60% feather hydrolysate + 40% peptone and 60% feather hydrolysate + 20% peptone + 20% BM while the least growth was observed in 100% BM. In the HNO _3_ feather hydrolysate media, following 100% peptone, the highest growth of *P. aeruginosa* was observed with 60% feather hydrolysate+ 40% BM, followed by 60% feather hydrolysate + 40% peptone, 60% feather hydrolysate + 20% peptone + 20% BM. The least growth was observed with 100% BM ( Figure 5).

### Growth rate of the *Pichia kudriavzevii*

**Figure 6.**
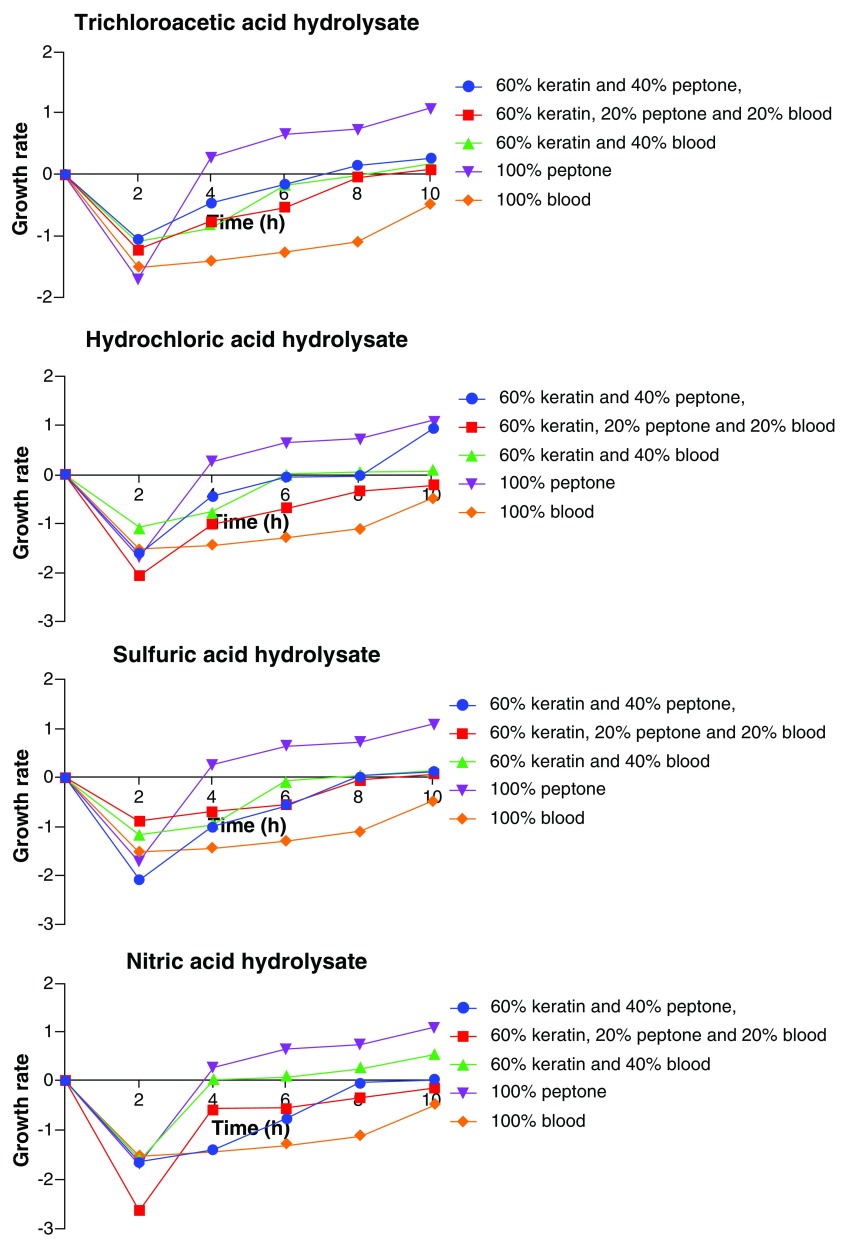
Growth rate of *Pichia kudriavzevii* in media with TCA, HCl, H _2_SO _4_ and HNO _3_ hydrolysates.

Figure 6 shows the growth pattern of *Pichia kudriavzevii* in the different media. *Pichia kudriavzevii* showed consistent increase in growth with the highest growth at the 10 ^th^ hour. The highest growth of the organism was 100% peptone media, while the least growth was observed in 100% BM. In the TCA feather hydrolysate media, the order of growth of *Pichia kudriavzevii* from the highest to the least in the media compositions was 100% peptone > 60% feather hydrolysate + 40% peptone > 60% feather hydrolysate + 40% BM > 60% feather hydrolysate, + 20% peptone + 20% BM >100% BM ( Figure 6).

In the HCl feather hydrolysate media, the order of growth was: 100% peptone > 60% keratin + 40% peptone > 60% feather hydrolysate + 40% BM > 60% feather hydrolysate + 20% peptone + 20% BM > 100% BM. In the H _2_SO _4_ feather hydrolysate media, the growth of the organism followed the order: 100% peptone > 60% feather hydrolysate + 40% BM > 60% feather hydrolysate + 40% peptone > 60% feather hydrolysate + 20% peptone + 20% > 100% BM. In the HNO _3_ feather hydrolysate media, the order of growth in the media compositions was: 100% peptone > 60% feather hydrolysate + 40% BM > 60% feather hydrolysate + 40% peptone > 60% feather hydrolysate + 20% peptone + 20% BM > 100% BM ( Figure 6).

### Growth rate of the *Proteus mirabilis*

**Figure 7.**
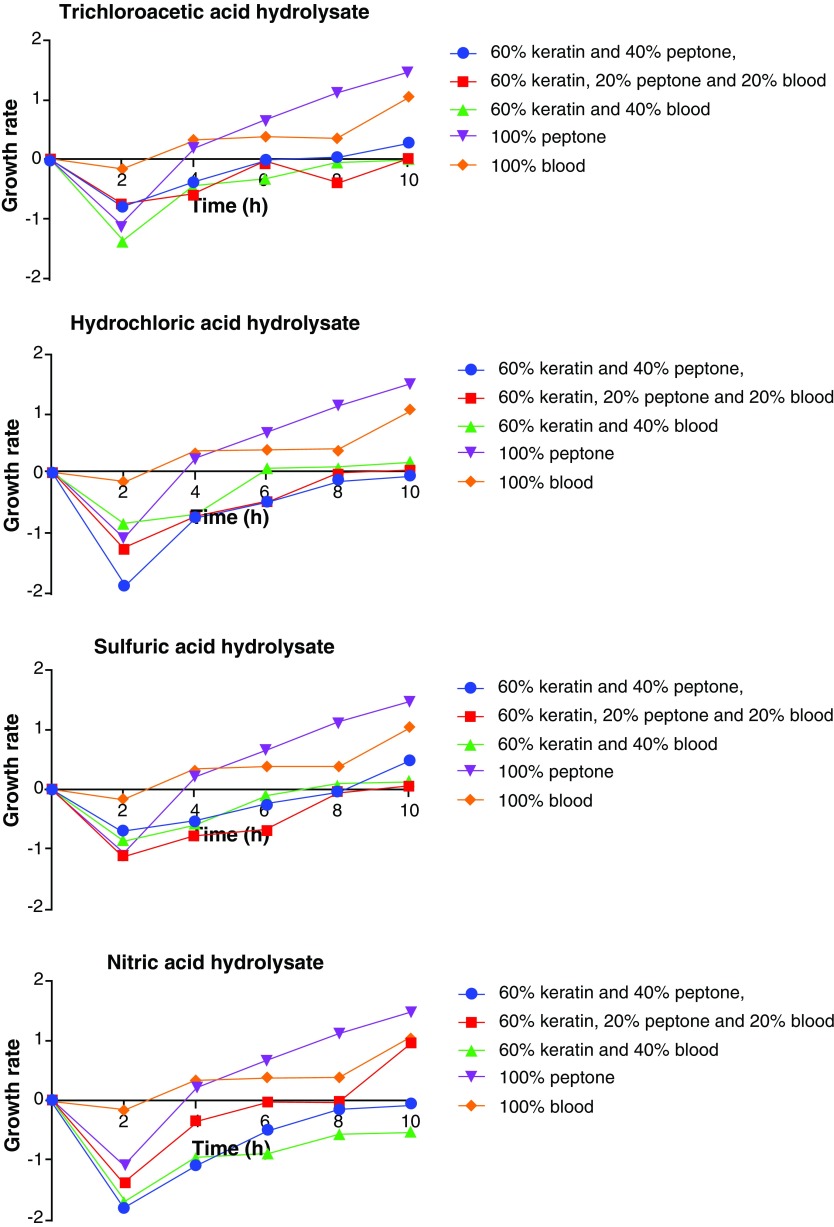
Growth rate of *Proteus mirabilis* in media with TCA, HCl, H _2_SO _4_ and HNO _3_ hydrolysates.

The growth rate of the *Proteus mirabilis* in the different media is shown in Figure 7. As shown in the Figure, the highest growth was recorded at the 10 ^th^ hour for all the media compositions against the respective acid hydrolysates.

In the TCA feather hydrolysate media, besides 100% peptone, in which the highest growth was recorded, the second highest growth of *Proteus mirabilis* was with 100% BM, followed by 60% feather hydrolysate + 40% peptone, 60% feather hydrolysate + 40% BM and the least growth was with 60% feather hydrolysate + 20% peptone + 20% BM. In the HCl feather hydrolysate media, after 100% peptone, the highest growth of *Proteus mirabilis* was observed with 100% BM, followed by 60% feather hydrolysate + 40% BM, 60% feather hydrolysate + 20% peptone + 20% BM, and the least growth was with 60% feather hydrolysate + 40% peptone ( Figure 7).

In the H _2_SO _4_ feather hydrolysate media, after 100% peptone, *Proteus mirabilis* growth pattern followed the order 100% peptone > 60% feather hydrolysate + 40% peptone > 60% feather hydrolysate + 40% BM 60% feather hydrolysate + 20% peptone + 20% BM ( Figure 7).

### Growth rate of the *Staphylococcus aureus*

In presence of the different hydrolysates, *Staphylococcus aureus* showed consistent increase in growth throughout the period of incubation. The 100% peptone media produced the best growth against all the acid hydrolysate media, while the least growth of the organism varied with media compositions.

**Figure 8.**
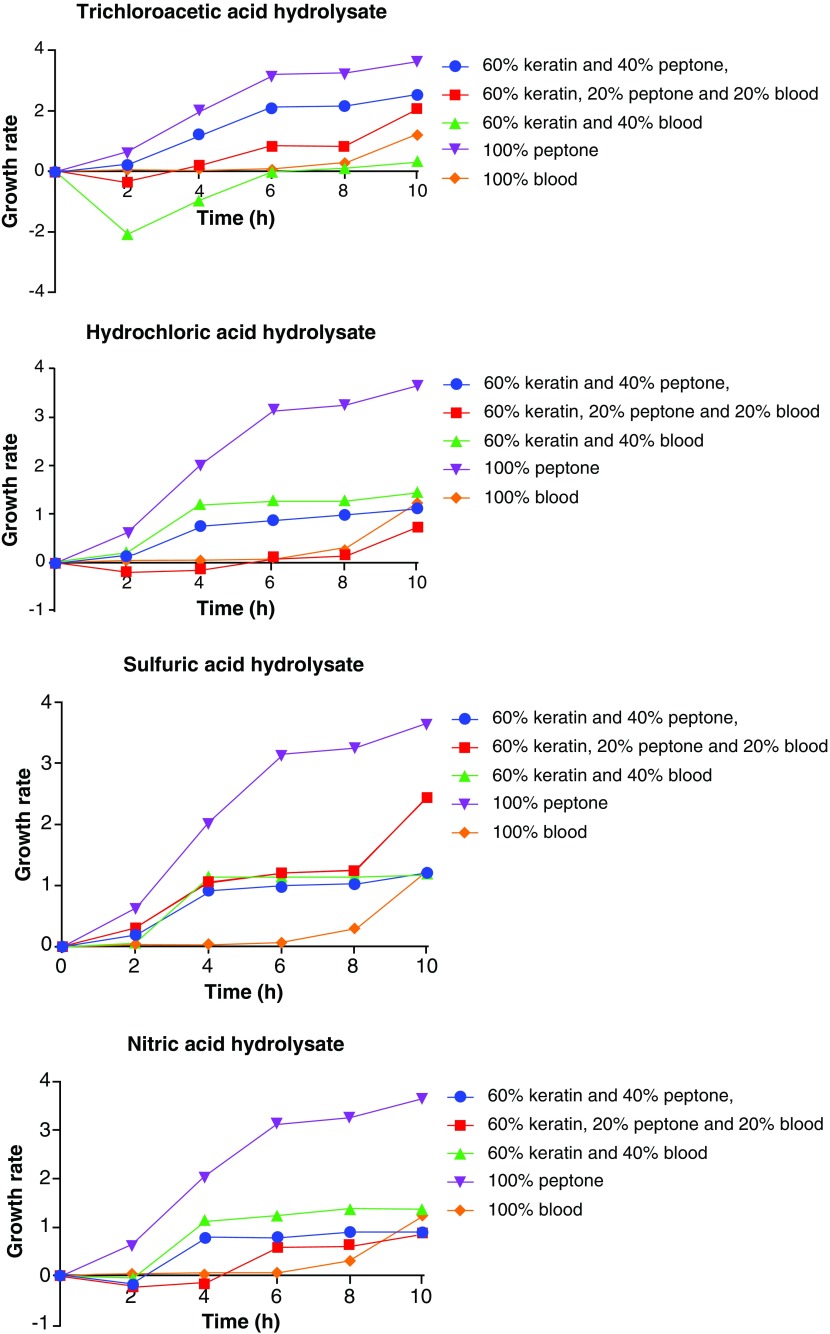
Growth rate of *Staphylococcus aureus* in media with TCA, HCl, H _2_SO _4_ and HNO _3_ hydrolysates.

In the TCA feather hydrolysate media, the 60% feather hydrolysate + 40% peptone media was second to the 100% peptone media in supporting the growth of *Staphylococcus aureus*, followed by 60% feather hydrolysate + 20% peptone + 20% BM, 100% BM and 60% feather hydrolysate + 40% BM in that order. In the HCl feather hydrolysate media, the second highest growth of the *Staphylococcus aureus* was observed with 60% feather hydrolysate + 40% BM, followed by 100% BM, 60% feather hydrolysate + 40% peptone, and 60% feather hydrolysate + 20% peptone + 20% BM in that order ( Figure 8).

In the H _2_SO _4_ feather hydrolysate media, the 60% feather hydrolysate+ 20% peptone + 20% BM ranked second to the 100% peptone media in the observed growth pattern for *Staphylococcus aureus*, followed by 60% feather hydrolysate + 40% BM, 60% feather hydrolysate + 40% peptone, and 100% BM in that order. In the nitric acid feather hydrolysate media, the growth pattern observed for *Staphylococcus aureus* followed the order 100% peptone > 60% feather hydrolysate + 40% BM > 60% feather hydrolysate + 40% peptone > 60% feather hydrolysate + 20% peptone + 20% BM ( Figure 8).

## Discussion

In this study, the chicken feather keratin peptone was obtained by alkaline hydrolysis and acid neutralization and precipitation delete. In most cases KOH, NaOH and Ca(OH) _2_ are used for the hydrolysis. Taskin *et al*., (2016) used the alkaline hydrolysis method with KOH in a study where peptone was obtained from sheep wool protein hydrolysate. From investigations, alkaline hydrolysis was reported to be able to produce a high yield of keratin and also enhance the keratin extraction effectiveness ( Sinkiewicz *et al*., 2017). Alkaline hydrolysis has also been studied and proven to be effective in the degradation of waste containing keratin and collagen ( Gousterova *et al*., 2005). It is opined that the use of alkaline hydrolysis inactivates of pathogens and prions such as transmissible spongiform encephalopathy (TSE) and bovine spongiform encephalopathy (BSE). The use of alkaline hydrolysis yields a BSE and TSE free hydrolysate media ( Matthews & Cooke, 2003). Despite the fact that heat is employed in most chemical hydrolysis processes to improve yield, the alkaline hydrolysis in this study was carried out under room temperature for approximately 10 h. Chemical hydrolysis conducted at high temperatures is said to lead to the destruction of amino acids ( Sinkiewicz *et al*., 2017).

Although acid hydrolysis has also been used in some studies to obtain protein hydrolysates ( Wisuthiphaet & Kongruang, 2015), it is argued that it could result in destruction of essential amino acids, such as methionine, cystine, cysteine and tryptophan, and conversion of glutamine and aspargine to glutamic and aspartic acid, respectively. It is indicated that during acid hydrolysis, the salts in hydrolysates can be injurious to the growth of the microorganisms ( Bucci & Unlu, 2000).

This study aimed to assess a cheaper source of protein for microbial culture than conventional nutrient media. Chicken feathers were chosen as the material for research because of its abundance, cost-efficiency and high protein content. The results obtained in this study with reference to the performance of the organisms in the formulated media compositions were viewed in comparison with the performance of the organisms in the commercially produced peptone. The comparison of the growth rate of the organisms in the different media with that in peptone was deliberate. Studies have revealed peptone as an excellent nitrogen and protein source for growing microorganisms and manufacture of growth media ( Markings, 2018).

The results of the present experiment showed that in most cases, the organisms growth was highest with the 100% commercially produced peptone while in other cases, various media compositions yielded higher growth of the organisms even better than in the 100% peptone.

The media compositions that favored the highest growth of the organism differed with each acid hydrolysate for every organism. The best growth of *Candida carpophila* was recorded with the commercial peptone irrespective of the acid hydrolysate media composition used. However, in the case of *Candida tropicalis*, the formulated media compositions from the chicken feather hydrolysate were able to yield higher growths of *Candida tropicalis* even better than the commercial peptone but only in the TCA hydrolysate, while the commercial peptone yielded the highest growth of the organisms in the other acid hydrolysates. *Pseudomonas aeruginosa*, *Pichia Kudriavzevii*, *Proteus mirabilis* and *Staphylococcus aureus* consistently had their highest growth with the commercial peptone irrespective of the acid hydrolysate used. In *Escherichia coli* and *Klebsiella pneumoniae*, variation of the highest growth with the hydrolysate media compositions and growth yield of the organisms higher than in the commercial peptones persisted. The variations in the media compositions that showed the best growth of the organisms were indications that the acids used in precipitating the keratin from the chicken feathers had significant impact on the efficacy of the media compositions. The optimum concentration of chicken feather keratin concentration that yielded maximum microbial growth was 60% feather hydrolysate + 40% peptone in *Escherichia coli*.

The ideal chicken feather hydrolysate that yielded microbial growth was the TCA hydrolysate. In a study by Taskin *et al*. (2016), using sheep wool protein hydrolysate as a peptone source for microorganisms, *Staphylococcus aureus* was observed to show poor growth performance in the media. However, growth performances of *Saccharomyces cerevisiae, Bacillus subtilis* and *Penicillium chrysogenum* were observed to be moderate in wool protein. Generally, the present study revealed that growth rate of the respective organisms varied from one media composition to the other. This observation has been reported by earlier workers ( Aspmo *et al*., 2005; Jones & Fayerman, 1987).

## Conclusion

Waste chicken feathers that were previously discarded and viewed as a burden to the environment can now be viewed as an important bio-resource and can be manipulated and explored widely as a biotechnological material. The study was able to utilize waste chicken feathers as a substitute for peptone in microbial culture.

In the study, the potential of the chicken feather keratin to support the growth of both bacteria and fungi was established. Chicken feather hydrolysate was also proven to be an excellent substrate particularly for the growth of *Escherichia coli*. Using a suitable process of hydrolysis, peptone from chicken feather keratin can be re-modified, industrialized and produced in bulk on a commercial scale. This research work could serve as precursor to exploring many other waste materials of high protein content which could be of biotechnological value.

## Data availability

The raw absorbance values from analysis of microbe growth using different growth media are available on figshare. DOI: https://doi.org/10.6084/m9.figshare.7376564.v1 ( Akpor *et al.* 2018b).

Data are available under the terms of the Creative Commons Attribution 4.0 International license (CC-BY 4.0).
