# Supplementary material for: Chicken feather hydrolysate as alternative peptone source for microbial cultivation
Source: F1000Res. 2019 Aug 7;7:1918. Originally published 2018 Dec 10. [Version 3] doi: 10.12688/f1000research.17134.3 (PMC6707403; doi:10.12688/f1000research.17134.3)
Supplement: Peer review history

# Peer review: Reviewer response for version 3

**DOI:** 10.5256/f1000research.22162.r52181

**Published:** 2019-08-16

**Authors:** Pius Okiki

**Affiliations:** 1 Department of Biological Sciences, Afe Babalola University, Ado Ekiti, Nigeria

I confirm that I have read this submission and believe that I have an appropriate level of expertise to confirm that it is of an acceptable scientific standard.

# Peer review: Reviewer response for version 2

**DOI:** 10.5256/f1000research.19748.r49287

**Published:** 2019-07-01

**Authors:** Pius Okiki

**Affiliations:** 1 Department of Biological Sciences, Afe Babalola University, Ado Ekiti, Nigeria

**Abstract**

 The second sentence under background should read: "Livestock waste, in form of poultry feather and cattle blood, are of value ..."

 The 1st sentence under subsection methods should read: "The growth of selected bacteria and fungi were monitored in different media prepared from varied concentrations of peptone, CFPH, ..."

 Nitric acid was written as nitic acid throughout the manuscript. This should be corrected.

**Methods** 

 Reference citation should be provided for preparation of blood meal from cattle blood (one is provided below ^1^), preparation of feather keratin hydrolysate and the microbial growth studies.

 The authors need to state where the bacteria and fungi used were obtained from; e.g. from the stock culture of the Microbiology Laboratory of their University, how the organisms were previously stored and if they were sub-cultured on agar plates before introduced into broth.

 The optimal growth temperature for pathogenic microorganisms (bacteria and fungi) is 37°C and not 24°C. This should be corrected.

 Statistical analysis: There was no evidence that LSD post hoc multiple comparison test was carried out. The growth performance of each microorganism tested in each formulated growth medium should be compared with that of standard peptone broth, and where there is no significant difference, such medium can easily replace peptone water.

**Results**

 The results are well written, but statistical comparison of the growth rates of the microorganisms in the formulated media with those of peptone broth will make the results better presented.

 In many places in the manuscript, medium was used in plural form as against media. This should be corrected.

**Discussion**

 Should take into consideration the results obtained from statistical comparison of the growth rates of the microbes in the formulated media with that of standard peptone broth.

**Conclusion**

 The 2nd sentence in the 1st paragraph could be written as: "The was able to utilize waste chicken feathers as a substitute for peptone in microbial culture". The 3rd sentence may not be necessary.

 The second paragraph should be re-written. "Following a thorough statistical analysis, the chicken feather hydrolysate may show excellent growth performances for other microorganisms apart from *E. coli*".

I confirm that I have read this submission and believe that I have an appropriate level of expertise to confirm that it is of an acceptable scientific standard, however I have significant reservations, as outlined above.

## References

[1] : Evaluation of methionine-fortified blood meal as substitute for fish meal in poultry broiler diets. *Afr. J. Poult. Farming*.2018;6(1) :216-224

# Response

**Published:** 2019-08-01

**Authors:** Oghenerobor Akpor

**Affiliations:** Landmark University, Nigeria

**Abstract**

 All the corrections suggested in the abstract are accepted and have been effected in the manuscript.

**Methods** 

 All corrections as indicated by the reviewer were attended to except the one on statistical analysis. The authors disagree with the reviewer on the lack of evidence of LSD post hoc multiple comparison test. d.

**Results**

 Corrections as indicated by the reviewer were effected except the ones on statistical analysis which the authors disagree with

**Conclusion**

 All the indicated corrections were effected in the manuscript

# Peer review: Reviewer response for version 2

**DOI:** 10.5256/f1000research.19748.r43782

**Published:** 2019-02-27

**Authors:** Adriano Brandelli

**Affiliations:** 1 Laboratório de Bioquímica e Microbiologia Aplicada, Universidade Federal do Rio Grande do Sul, Porto Alegre, Brazil

The manuscript was modified addressing only the comments from Reviewer #1, but none of the points raised in my review have been modified to date. Thus, I have no additional comments to make on the article until the authors provide a detailed response to the points raised in the initial review.

I confirm that I have read this submission and believe that I have an appropriate level of expertise to state that I do not consider it to be of an acceptable scientific standard, for reasons outlined above.

# Peer review: Reviewer response for version 2

**DOI:** 10.5256/f1000research.19748.r43783

**Published:** 2019-02-06

**Authors:** Swati Sharma

**Affiliations:** 1 Faculty of Chemical Engineering and Natural Resources, Universiti Malaysia Pahang, Pahang, Malaysia

The manuscript has been edited as per the suggestions.

I confirm that I have read this submission and believe that I have an appropriate level of expertise to confirm that it is of an acceptable scientific standard.

# Peer review: Reviewer response for version 1

**DOI:** 10.5256/f1000research.18732.r42593

**Published:** 2019-01-29

**Authors:** Adriano Brandelli

**Affiliations:** 1 Laboratório de Bioquímica e Microbiologia Aplicada, Universidade Federal do Rio Grande do Sul, Porto Alegre, Brazil

Poultry industry experienced a constant growth during the last decades, generating an increased amount of waste, in the form or feathers, viscera, skin, and dead-on-arrival (Brandelli et al. 2015 ^1^). Alternatives for the correct management of such poultry waste have been evaluated. Conversion of feathers into hydrolysates is a possible alternative to produce ingredients for feed, fertilizers, and cultivation media formulations (Daroit and Brandelli 2014 ^2^). This topic merits investigation, since the correct management of such waste material has great environmental relevance. In addition, it could represent an added value to the industry.

 In the current article, feathers were hydrolyzed by chemical methods and the resulting hydrolysates were tested as a peptone source for microbial growth. The utilization of byproducts obtained from abattoir as ingredients for culture media has been previously described. In particular, waste feathers from poultry processing industry have been converted as peptone source for bacterial growth (Taskin and Kurbanoglu, 2011 ^3^) and for development of culture media for production of mosquitocidal bacteria (Poopathi and Abidha, 2007 ^4^). More detailed studies include the use of feather peptone for microbial production of carotenoids (Taskin et al. 2011 ^5^) and protein hydrolysates (Kshetri, 2017 ^6^). Therefore, the results presented in this work are rather confirmatory, representing a restricted contribution to the field. Additional data should be included to improve this article. In this present form, the manuscript contains preliminary data and lacks sufficient novelty to be considered for indexing.

 In addition, a limited number or microorganisms was tested. The selection criteria is not clearly described and should be justified. It seems difficult to take solid conclusions since the microbial species tested are limited and only a single strain of each species was tested. Considering that nutritional requirements can be very variable depending on the microorganism, the inclusion of fastidious microorganisms, lactic acid bacteria, and filamentous fungi should be considered.

 Some points on Methods section need to be revised as follows:

- Feathers should be rinsed with abundant water to remove excess detergent before further treatment. Residual detergent could be toxic for some microorganisms.
- The hydrolysates were air-dried. The reason for not using the oven is unclear since this method was used for blood meal. In this regard, blood meal is mentioned in the Abstract but was not considered in the Discussion. Why was cattle blood used instead of chicken blood?
- The origin of microbial species tested should be clearly described. Available collection strains should be used in this type of study.
- The evaluation was performed in liquid medium, supposedly to quantify microbial growth. However, only absorbance at 750 nm (not 600 nm?) was measured, and viable cell counts were not determined. The evaluation of growth on agar plates could give interesting results.

Finally, the Results section is redundant, since it essentially consists a repetitive description of growth curves. Presentation of results should be carefully reconsidered.

I confirm that I have read this submission and believe that I have an appropriate level of expertise to state that I do not consider it to be of an acceptable scientific standard, for reasons outlined above.

## References

[1] : Microbial enzymes for bioconversion of poultry waste into added-value products. *Food Research International*.2015;73: 10.1016/j.foodres.2015.01.015 3-12 10.1016/j.foodres.2015.01.015

[2] : A current assessment on the production of bacterial keratinases. *Crit Rev Biotechnol*.2014;34(4) : 10.3109/07388551.2013.794768 372-84 10.3109/07388551.2013.794768 23937252

[3] : Use of waste chicken feathers as peptone for production of carotenoids in submerged culture of Rhodotorula glutinis MT-5. *European Food Research and Technology*.2011;233(4) : 10.1007/s00217-011-1561-2 657-665 10.1007/s00217-011-1561-2

[4] : Use of feather-based culture media for the production of mosquitocidal bacteria. *Biological Control*.2007;43(1) : 10.1016/j.biocontrol.2007.04.019 49-55 10.1016/j.biocontrol.2007.04.019

[5] : Evaluation of waste chicken feathers as peptone source for bacterial growth. *J Appl Microbiol*.2011;111(4) : 10.1111/j.1365-2672.2011.05103.x 826-34 10.1111/j.1365-2672.2011.05103.x 21762471

[6] : Transforming Chicken Feather Waste into Feather Protein Hydrolysate Using a Newly Isolated Multifaceted Keratinolytic Bacterium Chryseobacterium sediminis RCM-SSR-7. *Waste and Biomass Valorization*.2019;10(1) : 10.1007/s12649-017-0037-4 1-11 10.1007/s12649-017-0037-4

# Peer review: Reviewer response for version 1

**DOI:** 10.5256/f1000research.18732.r42168

**Published:** 2019-01-22

**Authors:** Swati Sharma

**Affiliations:** 1 Faculty of Chemical Engineering and Natural Resources, Universiti Malaysia Pahang, Pahang, Malaysia

**Abstract**

1. Give full forms of TCA...
2. .....also gave the best growth of *C. tropicalis *and *Klebsiella pneumoniae *respectively. In this sentence in what respect is respectively used?
3. In the manuscript total eight microorganisms were considered and conclusion is all about one microorganism. Justify?
4. Specify from where blood meal was taken.

**Introduction**

1. Keratin proteins are grouped into the alpha and beta keratins. Chicken feathers and feathers from most birds are composed majorly of the beta keratin. Provide with suitable reference.

**Methods**

1. Cleaning method of feathers was obtained from previous research. Need to cite the reference for the same
2. What was the basis to choose 1M NaOH for the study. Why didn't you try different concentrations? Is it standardized in your laboratory or taken from previous research, if so cite the reference.
3. On what basis were acids chosen for precipitation?
4. Heated at 60°CC for...... remove double C.
5. Naming should be same for the combination throughout the manuscript
6. Why didn't you try 100% feather hydrolysate?
7. Correct the mistake in sentence ed by 60% feather hydrolysate + 20% peptone + 20% blood, 60% feather hydrolysate + 40% peptone, and 100% blood in that order, while the least growth was observed with 60% feather hydrolysate + 40% blood....it should be Blood meal not blood in whole manuscript and same thing apply for feather hydrolysate
8. Give names to all medium like a, b...

**Results**

1. Overall the result section is good.
2. Conclusion is well written.  

I confirm that I have read this submission and believe that I have an appropriate level of expertise to confirm that it is of an acceptable scientific standard, however I have significant reservations, as outlined above.

# Response

**Published:** 2019-01-23

**Authors:** Oghenerobor Akpor

**Affiliations:** Landmark University, Nigeria

The authors appreciate thoroughness of the reviewer and the comments. Almost all the points raised that required modification have been attended to in the revised version. With respect to the use 1 M NaOH and the acids for hydrolysis and precipitation, respectively, that was ascertained during method development and the pilot phase of the study.
